# Dispersion as an Important Step in the *Candida albicans* Biofilm Developmental Cycle

**DOI:** 10.1371/journal.ppat.1000828

**Published:** 2010-03-26

**Authors:** Priya Uppuluri, Ashok K. Chaturvedi, Anand Srinivasan, Mohua Banerjee, Anand K. Ramasubramaniam, Julia R. Köhler, David Kadosh, Jose L. Lopez-Ribot

**Affiliations:** 1 Department of Biology and South Texas Center for Emerging Infectious Diseases, The University of Texas at San Antonio, San Antonio, Texas, United States of America; 2 Department of Biomedical Engineering, The University of Texas at San Antonio, San Antonio, Texas, United States of America; 3 Department of Microbiology and Immunology, The University of Texas Health Science Center at San Antonio, San Antonio, Texas, United States of America; 4 Division of Infectious Diseases, Children's Hospital, Boston, Massachusetts, United States of America; Washington University School of Medicine, United States of America

## Abstract

Biofilms are dynamic microbial communities in which transitions between planktonic and sessile modes of growth occur interchangeably in response to different environmental cues. In the last decade, early events associated with *C. albicans* biofilm formation have received considerable attention. However, very little is known about *C. albicans* biofilm dispersion or the mechanisms and signals that trigger it. This is important because it is precisely *C. albicans* cells dispersed from biofilms that are the main culprits associated with candidemia and establishment of disseminated invasive disease, two of the gravest forms of candidiasis. Using a simple flow biofilm model recently developed by our group, we have performed initial investigations into the phenomenon of *C. albicans* biofilm dispersion, as well as the phenotypic characteristics associated with dispersed cells. Our results indicate that *C. albicans* biofilm dispersion is dependent on growing conditions, including carbon source and pH of the media used for biofilm development. *C. albicans* dispersed cells are mostly in the yeast form and display distinct phenotypic properties compared to their planktonic counterparts, including enhanced adherence, filamentation, biofilm formation and, perhaps most importantly, increased pathogenicity in a murine model of hematogenously disseminated candidiasis, thus indicating that dispersed cells are armed with a complete arsenal of “virulence factors” important for seeding and establishing new foci of infection. In addition, utilizing genetically engineered strains of *C. albicans* (*tetO-UME6* and *tetO-PES1*) we demonstrate that *C. albicans* biofilm dispersion can be regulated by manipulating levels of expression of these key genes, further supporting the evidence for a strong link between biofilms and morphogenetic conversions at different stages of the *C. albicans* biofilm developmental cycle. Overall, our results offer novel and important insight into the phenomenon of *C. albicans* biofilm dispersion, a key part of the biofilm developmental cycle, and provide the basis for its more detailed analysis.

## Author Summary

*Candida albicans* is the main causative agent of candidiasis, a difficult-to-treat infection that occurs mostly in severely immunosuppressed and other at-risk patients. Candidiasis is often associated with the formation of biofilms (attached microbial communities encapsulated within a protective matrix) on host surfaces and/or implantable medical devices, most notably intravascular catheters. In recent years, for *C. albicans*, the process of biofilm formation has received much attention. However, the same is not true for biofilm dispersion (the release of cells from the biofilm). This is important since these dispersed cells are responsible for the subsequent establishment of disseminated candidiasis at distal organs. Here we have taken advantage of a model of biofilm formation under conditions of flow recently described by our group to study and characterize the phenomenon of *C. albicans* biofilm dispersion. Rather than an end-stage process, our results indicate that dispersion occurs at all different stages of the biofilm developmental cycle and is influenced by nutritional and other physiochemical conditions. In addition, our findings provide initial insights into how this process is regulated at the molecular level. We also demonstrate that dispersed cells display distinct phenotypic properties that are associated with increased virulence, with important clinical repercussions.

## Introduction

Candidiasis represents the third to fourth most frequent nosocomial infection in hospitals both in the US and worldwide and *C. albicans* remains its most frequent causative agent [1]–[4]. Despite recent advances in the management of these infections, and even after treatment with available antifungal drugs, the mortality rates for patients with candidiasis lie in the 30–50% range [5]–[7]. Since *C. albicans* forms robust, architecturally complex biofilms on implanted synthetic material *in vivo*, a variety of manifestations of candidiasis are associated with biofilm formation on medically required foreign bodies, most notably intravascular catheters but also peritoneal dialysis catheters, ventriculo-peritoneal shunts and other implanted devices necessary for patient survival and quality of life [8]–[10]. Yeast, mostly *C. albicans*, are the third leading cause of intravascular catheter-related infections, with the second highest colonization to infection rate and the overall highest crude mortality [11]. These biofilm infected catheters serve as reservoirs of infectious particles, releasing cells into the blood stream in which they gain access to distant sites with the potential for metastatic infection of deep organs. In patients without cancer, infected catheters are the most important source of life-threatening blood stream infections and subsequent invasive disseminated candidiasis, two of the gravest forms of disease mostly responsible for the high mortality rates. Removal of catheters is recommended for patients with *Candida* bloodstream infections to facilitate more rapid clearance of the bloodstream and better prognosis [5],[12],[13].

*C. albicans* biofilms have been developed on several different model systems *in vitro* and many experimental variables have been incorporated into these systems in order to mimic *in vivo* conditions in patients such as flow to simulate physiological blood flow, coating of substrate surfaces with various host blood and salivary proteins, variation in the nutrient composition of the media employed and use of common device materials [8], [14]–[20]. These models have enabled extensive study, particularly of the early phases of *C. albicans* biofilm biology. It is now well established that a *C. albicans* biofilm lifecycle begins when planktonic yeast cells adhere to a substrate and begin to proliferate, ultimately leading to the formation of a highly structured mature biofilm comprised of complex intertwining layers of yeast, pseudohyphae and hyphae embedded in extracellular matrix. The cycle is thought to continue with biofilm dispersion/detachment or dissolution, when cells released from the biofilm seed new surfaces, thus completing the biofilm life-cycle [10],[21],[22].

While numerous studies have explored and are starting to unravel the molecular mechanisms underlying the initial stages of biofilm formation and development [14], [23]–[25], the events associated with dispersion of cells from *C. albicans* biofilms have received little attention. This is despite the implication that dispersion of cells from biofilms may be responsible for device associated candidemia and subsequent disseminated invasive disease. The bacterial biofilm literature has emphasized the role of nutrition in inducing biofilm dissolution: in general, biofilm dispersion occurs in response to environmental changes such as lack or depletion of nutrients or a change in the nutrient composition. A variety of chemical and enzymatic treatments can also be used to cause significant dispersion from bacterial biofilms [26],[27]. Several reports have elucidated some of the molecular mechanisms orchestrating dispersion in bacterial biofilms [28]–[31]. In *C. albicans*, it has been speculated, although not directly tested, that nutrient limitation in a mature biofilm may lead to quorum sensing-mediated dispersion of daughter cells less adherent to the biofilm matrix [22]. It is also theorized that the yeast cells in the biofilm might possess mechanisms that enable them to regulate the activity of their adhesins, thereby allowing them to become released from the overall biofilm structure [32]. A more direct study has been recently reported on *C. albicans* biofilm detachment rather than biofilm dispersion. The study describes development of biofilms on silicon elastomer tubing at very high shear forces and the massive detachment event of early stage biofilms in a rich medium, after draining the tubing [33]. Differential gene expression analyses revealed that this early detachment episode was not orchestrated at the level of transcriptional regulation, and was only coordinated by a small population of surface adhered cells [33].

The aim of our present study is to present evidence that *C. albicans* biofilm dispersion can be triggered by changes in the physiochemical environment, specifically changes in the concentration of various carbon sources and growth conditions. In addition, in this study, we provide evidence for the fact that *C. albicans* biofilm dispersion involves phenotypic modifications of the dispersing cells, with important implications for virulence. We also substantiate that biofilm dispersion to a large extent can be regulated by manipulation of levels of expression of key morphogenetic genes.

## Materials and Methods

### Ethics statement

All animal experimentation was conducted following the National Institutes of Health guidelines for housing and care of laboratory animals and performed in accordance with Institutional regulations after pertinent review and approval by the Institutional Animal Care and Use Committee at The University of Texas at San Antonio. Human Umbilical Cord Vein Endothelial Cells (HUVEC) were obtained from discarded umbilical cords; because these are considered pre-existing clinical samples and there were no patients' identifiers, the use of these samples is considered exempt under the Department of Health and Human Services (DHHS) Policy for Protection of Human Research Subjects, as well as by the Institutional Review Board at The University of Texas at San Antonio.

### *C. albicans* strains and media

The strains used in this study were *C. albicans* strains SC5314 (wild type) and the tetracycline regulatable *tetO-PES1*, and *tetO-UME6* strains that have been previously described [34],[35]. Stock cultures were stored in 15% glycerol at −80°C. Strains were routinely grown under non-filament-inducing conditions (media at 30°C) in the presence or absence of 25 µg/ml of doxycycline (DOX). Dilutions (1∶1 unless otherwise indicated) of YPD (0.5% yeast extract, 1% bacto peptone, 1% glucose), YNB (BD Biosciences, San Jose, CA) with 50 mM glucose or RPMI (Sigma, St. Louis, MO) with MOPS buffer were used for culturing both biofilm and planktonic cultures.

### Biofilm culture conditions

Biofilms were cultured in a simple flow biofilm model described recently by our group [20]. This model involves a controlled flow of fresh medium via Tygon tubing (Cole-Parmer, Vernon Hills, IL) into a 15 ml polypropylene conical tube (BD, Franklin, NJ) holding a silicon elastomer (SE) strip. Medium flow is controlled by connecting the tubing to a peristaltic pump (Masterflex L/S Easy-Load II, Cole-Parmer). The whole apparatus was placed inside a 37°C incubator to facilitate biofilm development at a controlled temperature. SE strips (1×9 cm_,_ Cardiovascular instrument Corp, Wakefield, MA), were sterilized by autoclaving and pre-treated for 24 hr with bovine serum. *C. albicans* was grown overnight at 30°C, washed, and diluted to an optical density at 600 nm (OD_600_) of 0.5 in the respective media. The SE strips were incubated with the diluted *C. albicans* suspension at 37°C for 90 min at 100 rpm agitation for the initial adhesion of cells for 90 min. Next, the strip was inserted into the conical tube and the peristaltic pump was turned on. The media flow rate was maintained at 1 ml/min.

### Estimation of shear stress

Biofilms in the flow biofilm model were subjected to a steady, laminar flow of medium at a rate of 1 ml/min. For a free-surface laminar flow over an inclined plane, the film thickness of the media on top of the biofilm, and the shear stress on the surface of the biofilm due to flow can be calculated from Bird et al., 1960 [36].
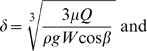





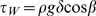



Where *δ* is liquid film thickness (mm), μ is dynamic viscosity of media ( = 0.7 cP), *ρ* is density of media ( = 1 g/ml), *W* is the width of the biofilm ( = 1 cm), *g* is gravitation constant ( = 9.8 m/s^2^), *Q* is flow rate ( = 1 ml/min), and *β* is the angle of inclination ( = 45°).

Substituting these values, we get, film thickness of media *δ* = 0.088 mm, and shear stress on the surface of the biofilm as 4.52 dyn/cm^2^.

### Processing of cells dispersed from biofilms and induction of biofilm dispersion

Standard growth conditions for biofilm development used media containing 50 mM glucose for 24 hours. At various time points during biofilm development, cells released from the biofilm in the flow-through were collected from the bottom of the conical tube. The dispersed cells were enumerated by hemocytometer and by colony counts on solid media (YPD plates). The dispersed cells were also visualized by light microscopy (Westover Scientific, Mill Creek, WA) and images processed by using Micron software (Westover Scientific, Mill Creek, WA). In some cases, the topmost layer of biofilms were tweezed with sharp tipped forceps and visualized under the microscope to gain an understanding of the process of dispersion.

For induction of dispersion, biofilms were pregrown for 24 hr in YNB medium (pH 7.2) containing 50 mM glucose. After 24 hr of biofilm growth, biofilm dispersion was induced by a sudden 10-fold increase or decrease in glucose concentration in the growth medium. In addition, dispersion was induced through addition of high concentrations of alternative carbon sources such as galactose (500 mM) and maltose (500 mM) to the growth medium. Dispersion was also induced when starvation was induced by treatment of preformed biofilms with sterile PBS, or by a change in pH of the medium (increase in pH from 7.2 to 9 or decrease to pH 3). Dispersion was indicated by an increase or decrease in the number of cells released in the flow-through at various time points during the experimental process.

For the study involving farnesol, farnesol was added to the reservoir medium to a concentration of 30 µM, 100 µM or 300 µM and the compound-containing medium was allowed to flow over 24 hr old preformed biofilms. Farnesol-mediated effects on dispersion were measured by cell counts at various time points (30 min, 90 min, 3 hr, 6 hr and 24 hr).

Manipulation of levels of gene expression in the *tetO-UME6* and *tetO-PES1* strains and the subsequent impact on biofilm dispersion was studied by the incorporation or omission of DOX (20 µg/ml) into the reservoir medium flowing over the biofilms. For these experiments the flow rate of the medium was always maintained at 1 ml/min.

### Scanning electron microscopy

Biofilms were placed in fixative (4% formaldehyde v/v, 1% glutaraldehyde v/v in PBS) overnight. The samples were rinsed in 0.1 M phosphate buffer and air dried in desiccators. To retain the original structure of the biofilms as much as possible, sample processing did not include harsh dehydration steps. The samples were coated with gold/palladium (40%/60%) and observed in a scanning electron microscope (Leo 435 VP) in high vacuum mode at 15 kV. The images were processed for display using Photoshop software (Adobe, Mountain View, CA.).

### Assessment of phenotypic properties (adhesion, biofilm formation) of dispersed cells

Both adhesion and biofilm formation assays were preformed in 96-well microtiter plates. Cells dispersed from 24 hr biofilms grown in YNB, were washed once and diluted to a cell concentration of 2×10^6^ cells/ml in YNB medium. Matched planktonic cells grown for 24 hr at 37°C in the same medium were also similarly processed. Aliquots of the diluted suspensions (2×10^5^ cells) were added into wells of the microtiter plates and incubated at 37°C. After 90 min., the wells were washed twice with sterile PBS and the extent of cellular adhesion to the wells was quantified by the XTT assay as described earlier [37]. Alternatively, the washed wells were treated with 150 µl of fresh YNB medium and further incubated for 24 hr at 37°C to promote biofilm formation. Biofilms were washed twice with sterile PBS and the extent of biofilm formation estimated using the semi quantitative XTT reduction assay.

### *C. albicans*/endothelial cell adhesion and invasion assays

Human Umbilical Cord Endothelial Cells (HUVEC) obtained from discarded umbilical cords were used within first three to five passages for all experiments. The cells were isolated following an established protocol [38] except that 0.5 mg/ml collagenase was used for 10 min. Briefly, the cords were washed with 20 ml Dulbecco's PBS (DPBS), and the cells were extracted from the cord by incubating with 0.5 mg/ml collagenase for 10 min at 37 C followed by DPBS wash. The isolated cells were centrifuged, resuspended in complete HUVEC media, and seeded into 75 cm^2^ flasks for culture. The media was changed the next day. The HUVEC culture media was prepared from M199 with sodium bicarbonate, supplemented with 20% FBS, 2 mM L-glutamine, 25 mM HEPES, 10 mg/ml Heparin sodium salt, 1% penicillin-streptomycin (all from Sigma), and Endothelial Cell Growth Supplement (ECGS; Biomedical Technologies, MA). The HUVECs were grown to confluence (2×10^6^ cells in 35 mm plates and 1×10^5^ cells in 96-well microtiter plates) and used for adhesion and invasion experiments. On the day of the experiment, the tissue culture medium above the endothelial cells was aspirated and replaced with fresh medium containing *C. albicans*, including either cells dispersed from biofilms or age-matched planktonic cells grown at 37°C. In all experiments, the ratio of *C. albicans* to endothelial cells was 1∶1. All incubations were performed for 90 min at 37°C in 5% CO_2_. Each experiment was repeated at least two times, using endothelial cells from different umbilical cords. After incubation, the plates were washed three times with sterile PBS, the washes pooled and cell numbers from each condition enumerated by hemocytometer and colony counts on YPD plates. Additionally, number of *C. albicans* cells adhered to the endothelial monolayer was counted under an inverted light microscope. At least 10 fields were randomly counted for each experimental condition. These fields were next enumerated for proportion of cells germinated versus non-germinated blastospores or pseudohyphal cells. A statistical analysis (ANOVA, p<0.05) was performed to analyze differences in adhesion between the two experimental conditions.

The ability of *C. albicans* to damage human vascular endothelial cells was assessed by the CytoTox-96 assay (Promega, Madison, WI), which measures the release of lactate dehydrogenase (LDH) from dying cells. For these experiments, planktonic and biofilm dispersed cells (as above) were diluted to various concentrations in HUVEC culture medium and were added to endothelial cells for 8 hr and 18 hr incubation times at 37°C in the presence of 5% CO_2_. The amount of LDH released from the co-culture system was quantified by spectrophotometry performed according to the manufacturer's instructions. Uninfected cultures (control 1) and *C. albicans* alone (control 2) incubated under identical conditions were included as negative controls. The total amount of LDH released was estimated by treating control uninfected endothelial cells with 9% Triton X-100 for 1 hr. The LDH released in the presence of *C. albicans* was quantified by using the following formula: [(experimental – control 1 – control 2)/(total – control 1)] ×100. The values were expressed as percentages of the total amount of LDH released.

### Assessment of virulence potential in a murine model of hematogenously disseminated candidiasis

The following two conditions of cell populations were used for the study: i) planktonic *C. albicans* cells grown for 24 hr in YNB medium incubated at 37°C, and ii) cells dispersed from medium, age and incubation temperature matched biofilms. The cells were spun down and washed twice in sterile pyrogen-free saline and counted by using a hemocytometer. After appropriate dilutions, 200 µl of cell suspension (corresponding to 2.8×10^5^ cells) were injected into the lateral tail vein of individual 6- to 8-week-old BALB/c mice (6 mice per group). Plate counts were carried out to confirm the number and viability of cells in the inoculum. Mice were monitored for survival for 21 days after infection (moribund animals were euthanized and recorded as dying on the following day). The Kaplan–Meier log rank test was used to determine any statistically significant differences between the groups infected with dispersed cells and planktonic cells respectively. This analysis was performed by using Prism and GraphPad software. After receipt mice were allowed a one week acclimatization period before experiments were started.

## Results

### Kinetics of *C. albicans* biofilm dispersion

**Figure 1:**
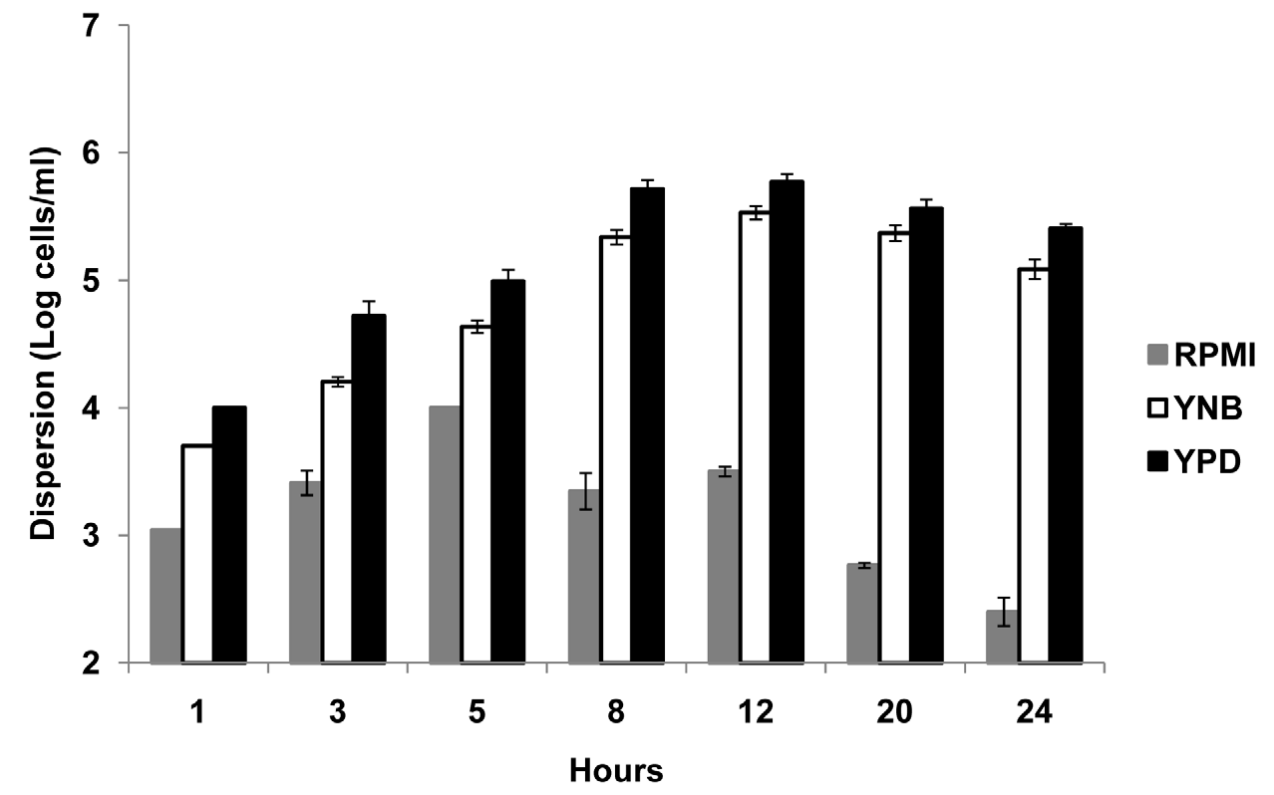
Quantification of numbers of dispersed cells from *C. albicans* biofilms grown in three different media. *C. albicans* biofilms were developed in RPMI, YNB and YPD media, and the number of dispersed cells recovered from the biofilms was counted over time. Results shown are expressed as mean and standard deviation from three independent experiments for each condition.

Silicon elastomer (SE) pieces were seeded with *C. albicans* and biofilms developed 24 hr under conditions of flow using three different media RPMI, YNB and YPD. Numbers of dispersed cells were enumerated at several time points during biofilm development (1, 3, 5, 8, 12, 20 and 24 hr) (Figure 1). Although in low numbers, dispersed cells were already present in the flow-through liquid collected during the early stages (1–3 hr) of biofilm development, irrespective of the media. The highest levels of dispersion were observed during the intermediate stages of biofilm formation, at a time where maximal proliferation of cells within the biofilm occurs [19],[21],[22]. Maximum numbers of cells were recovered from the flow-through medium after 5 hr of biofilm growth in RPMI, and 12 hr in YNB and YPD (Figure 1). As the biofilm reached maturity (over 20–24 hr), there was a significant decrease in cellular dispersion under all conditions tested. Overall, growth in YPD medium, nutritionally the richest of the three media used in this study, resulted in the largest amount of dispersion, at least two-fold higher than YNB at every time point tested (Figure 1); while biofilms grown in RPMI consistently yielded the lowest numbers of dispersed cells at all time points examined.

### *C. albicans* cells dispersed from biofilms are predominantly in the yeast form

*C. albicans* biofilms grown in several media are typically comprised of a mixture of yeast, pseudohyphae and hyphae, displaying a typical multilayered structure [10], [15], [19]–[22]. Using light microscopy, we examined the morphology of cells dispersed from biofilms developed for 24 hr under flow conditions, in RPMI, YNB and YPD medium (RPMI-grown biofilms are normally more “filamentous” compared to the more “yeasty” biofilms obtained when YNB or YPD are used). We discovered that regardless of the media used, the dispersed cells were predominantly in the yeast form. A large number of these dispersed yeast cells were unbudded and had an elongated morphology. Additionally, the differences in form of the yeast cells were largely dependent on the type of media used. In RPMI, a medium that favors hyphal growth, the dispersed cells comprised 92% of unbudded yeast cells and 86% of these were elongated in form (see images in Figure S1A and cell counts in Table S1). Biofilms grown in YNB (a synthetic defined medium) or YPD (an enriched medium favoring yeast growth) released a higher proportion of budding yeast cells compared to RPMI (Table S1). In fact, in YPD medium, the percentages of budded and unbudded yeast cells were similar. Apart from yeast cells, biofilms intermittently released a small number of pseudohyphal cells (Table S1). Under no conditions did we observe true hyphae being released from the biofilm biomass. When a portion of the biofilm surface was tweezed and examined under a light microscope, the dispersed yeast cells appeared to be released from the hyphal layers of the biofilm (Figure S1B). This was more evident when biofilms grown under flow conditions were examined by scanning electron microscopy (SEM). Large numbers of yeast cells were observed budding off from top-most hyphal layers of the biofilm (Figure S1C).

### The effectiveness of *C. albicans* biofilm dispersion is dependent on environmental growth conditions, including carbon source and pH

**Figure 2:**
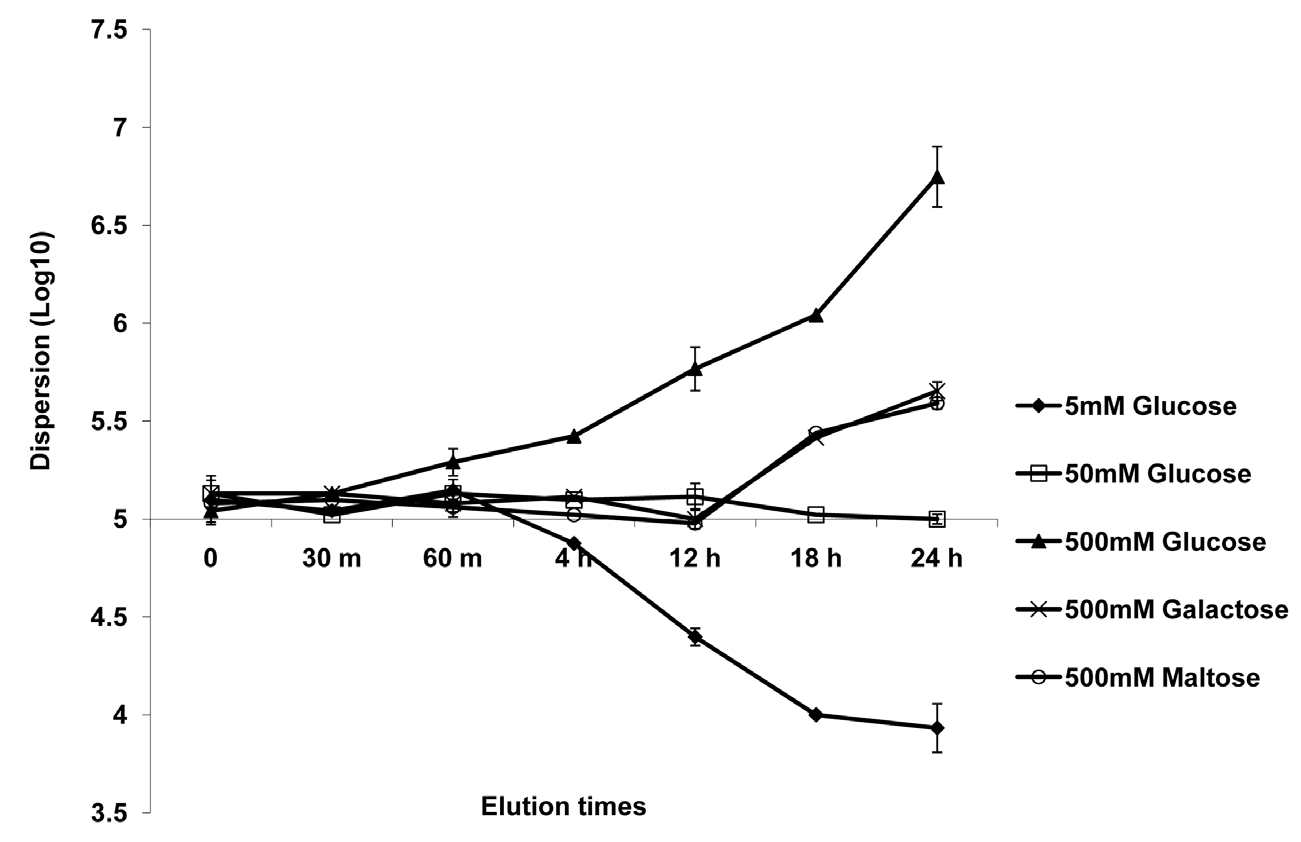
Effect of carbon source on *C. albicans* biofilm dispersion. Biofilms were developed for 24 h in YNB medium with 50 mM glucose. The media was then changed to YNB containing varying concentrations of glucose or alternative carbon sources (maltose and galactose). The impact of varying carbon sources and/or concentrations on the level of dispersion was quantified at various time points. Results shown are expressed as mean and standard deviation from two independent experiments for each condition tested.

Biofilm dispersion was monitored through the addition of alternate carbon substrates such as glucose, galactose or maltose to biofilm growth medium. First, biofilms were grown in YNB plus 50 mM glucose for 24 hr, and biofilm dispersion was quantified at this time point. The biofilms were then treated with YNB media containing 5 mM or 500 mM glucose, 500 mM galactose, or 500 mM maltose. Biofilms grown in 50 mM glucose were used as controls. Levels of dispersion were estimated by determining the cell number and viability counts of *C. albicans* in the flow-through liquid at several time points (ranging from 0 h to 24 hr) after the switch to the new media. All tested carbon sources were found to induce dispersion under high-nutrient conditions. Treatment with 500 mM glucose was most effective at inducing dispersion, resulting in a 2-fold increase in cell counts for dispersed cells as early as 60 min (Figure 2). By 24 hr, the biofilms released approximately 50–60 times more cells in the presence of 500 mM glucose than in 50 mM glucose (Figure 2). In fact, such a prolonged treatment with 500 mM glucose eventually led to complete biofilm dissolution within 30 hr (the biofilms appeared to dissolve and completely detach from the SE). An opposite effect was observed when dispersion was monitored after the switch to 5 mM glucose: this 10-fold reduction in glucose concentration resulted in approximately 5 and 12-fold decreases in dispersion at 4 hr and 24 hr, respectively (Figure 2). High concentrations of galactose and maltose (500 mM) generated comparable levels of biofilm dispersion throughout the 24 hr follow-up period. Dispersion in these two media was generally slow, with the first signs of significant increase (about 2-fold) seen only 12 hr after the switch. By 24 hr, the number of dispersed cells only doubled compared to cells dispersed from biofilms grown in 50 mM glucose (Figure 2).

In the same series of experiments, we included a condition in which the control biofilms (grown for 24 hr in 50 mM glucose) were subjected simply to sterile PBS, for 12 hr, to induce nutrient starvation. A greater than 20-fold decrease in the release of cells from the biofilm was seen as early as 2 hr, culminating in >50-fold decrease by the end of 12 hr (data not illustrated in figure).

Next, we tested the impact of changes in media pH on *C. albicans* biofilm dispersion. For this, biofilms were first developed for 24 hr in YNB (pH 7.2), and the number of dispersed cells were counted at this time point. The pH of the reservoir YNB medium was then either decreased to 3 or increased to 9, thereby subjecting the biofilms to an acidic or alkaline medium for another 24 hr. As shown in Figure S2A, a decrease in pH triggered a 5-fold increase in the number of dispersed cells as compared to the base line level. To the contrary, an increase in pH from 7.2 to 9 resulted in a 4-fold curtailment of biofilm dispersion (Figure S2A).

Biofilms developed for 24 hr were also exposed to different concentrations of farnesol, a quorum sensing molecule that inhibits filamentation and biofilm formation in *C. albicans* [39],[40], and the dispersed cells released from the biofilms as a result of this treatment were enumerated. Concentrations of farnesol up to 30 µM did not trigger an increase in dispersion from the preformed biofilms, even after 24 hr of treatment (Figure S2B). In fact, treatment with a higher concentration of farnesol (100 µM) also did not significantly alter the baseline rate of dispersion until 24 hr. After 24 hr, however, the rate of dispersion increased ∼3.5-fold compared to that released by 30 µM farnesol. A higher (definitely supraphysiological) farnesol concentration of 300 µM triggered a 6-fold increase in biofilm dispersion as early as 30 min. By 6 h, the dispersion rates more than doubled and remained steady until 24 hr. Between 24 – 30 hr, the biofilms treated with 300 µM farnesol appeared to dissolve and detach from the substrate. Similar to farnesol, physiological concentrations of tyrosol (50 µM) did not have an effect on biofilm dispersion (not shown).

### *C. albicans* dispersed cells display enhanced adhesion and ability to form biofilms

**Figure 3:**
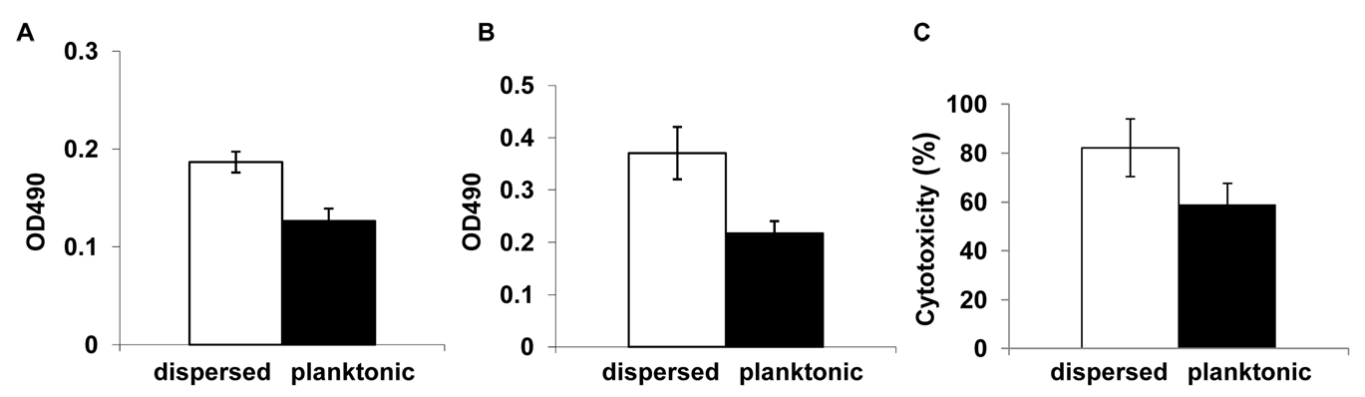
Evaluation of the adhesive, biofilm development and invasive properties of dispersed cells. The adhesive and biofilm-forming properties of planktonic *C. albicans* yeast cells grown at 37°C, and biofilm dispersed cells were compared in microtiter plate-based adhesion (A) and biofilm development (B) assays using XTT-reduction. Measurement of LDH released from endothelial cells damaged by *C. albicans* grown under planktonic conditions and by biofilm dispersed cells were expressed as percent cytotoxicity (C).

We entertained the idea that dispersed cells, originally a part of the biofilm biomass, may exhibit distinct phenotypic characteristics compared to cells in established planktonic cultures. Since dispersed cells were mostly in the quantifiable yeast form, their phenotypic properties could experimentally be compared to planktonic yeast cells. Thus, dispersed cells recovered from 37°C grown, 24 hr old biofilms were compared to age matched planktonic yeast cells for their ability to adhere to plastic and form biofilms. The adherence assays were carried out by seeding the wells of microtiter plates and incubating for 90 min at 37°C. Significantly more dispersed cells adhered to the wells compared to their planktonic counterparts (ANOVA, p≤0.01) (Figure 3A). We used a similar assay, to next compare the biofilm-forming abilities of the dispersed versus planktonic cells using the well-described 96 well static biofilm model. After 24 hr, dispersed cells were able to form more robust biofilms that displayed 41% higher metabolic activities (as determined by the XTT-colorimetric readings) compared to biofilms that originated from seeding regular planktonic cells (Figure 3B). Overall, dispersed cells were up to 1.67 fold more adherent and formed biofilms 1.8 fold better than planktonic cells.

### Dispersed cells from *C. albicans* biofilms display enhanced adhesion to endothelial cells and cause increased endothelial cell damage

Establishment of metastatic foci of infection of *Candida* species, a critical step in the pathogenesis of deep organ candidiasis, is presumably dependent on the fungus traversing the vascular endothelium [41],[42]. Hence, we expanded the *in vitro* adhesion assays to investigate *C. albicans*-endothelium attachment. *C. albicans* cells dispersed from biofilms, and age- and medium-matched planktonic cells grown at 37°C were allowed to adhere to endothelial cells for 90 min at 37°C. We found that dispersed cells adhered in larger numbers to the endothelial layer than their planktonic counterparts (ANOVA, p≤0.05) (Table S2). In fact, the pattern and percent increase in adhesion was comparable to the increased adhesion to plastic. We questioned if the differences in adherence were directly associated with differences in extent of filamentation by the *C. albicans* cell populations. Besides counting the number of cells adhered to the endothelial monolayer, we also scored for the proportion of yeast versus germ tubes and pseudohyphae in the adhered cell population. As shown in Table S2, cells released by biofilms were more likely to germinate at higher percentage rates than planktonic yeast cells recovered from growth at the same temperature. We subsequently tested the potential of each cell population to damage an endothelial cell monolayer. To quantify cell injury, we compared the levels of LDH released when planktonic *C. albicans* yeast cells (grown for 24 hr at 37°C) and cells dispersed from 24 hr biofilms were co-cultured with HUVEC. In general, cell damage was dependent on time of contact and infectivity rate (not shown). After 18 hr, biofilm dispersed cells exhibited significantly higher cytotoxicity than planktonic cells grown at 37°C (ANOVA, p≤0.01) (Figure 3C).

### *C. albicans* cells dispersed from biofilms display increased virulence in a murine model of hematogenously disseminated candidiasis

**Figure 4:**
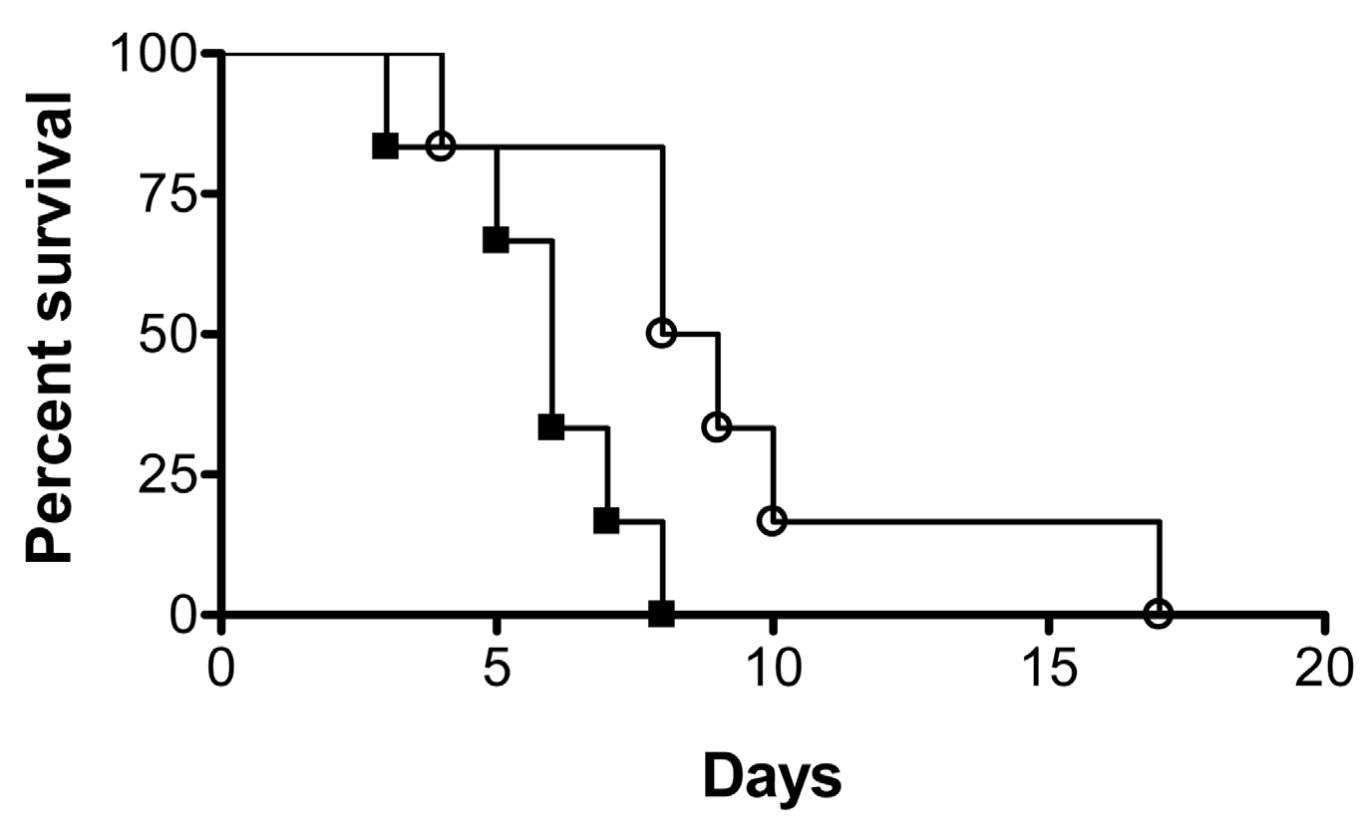
Dispersed cells display increased virulence in vivo. Groups of mice were injected with the same dose (2.8×10^5^ CFU) of either dispersed cells from biofilms (black squares) or cells obtained from matched planktonic cultures (open circles), and their survival was monitored over the course of infection in this murine model of hematogenously disseminated canididiasis. Statistically significant differences were observed between the corresponding survival curves generated for the two groups of mice (*P*<0.05).

Subsequent to biofilm formation on intravascular catheters, *C. albicans* biofilm dispersion is the main step associated with life-threatening clinical infections in susceptible patients since cells released from the biofilm are responsible for candidemia and dissemination to target organs. We hypothesized that dispersed cells may be more virulent than regular planktonic cells. We performed a side-by-side comparison of the pathogenicity of dispersed cells versus matched planktonic cells using the well established murine model of hematogenously disseminated candidiasis. Results of this series of experiments indicated that mice infected via the tail vein with dispersed cells from biofilms succumbed to the infectious challenge faster than mice injected with matched yeast cells that had been grown planktonically prior to inoculum preparation (P<0.05) (Figure 4). Thus, in this model dispersed cells show increased virulence compared to their planktonic counterparts.

### Manipulation of levels of *UME6* and *PES1* gene expression affect *C. albicans* biofilm dispersion

**Figure 5:**
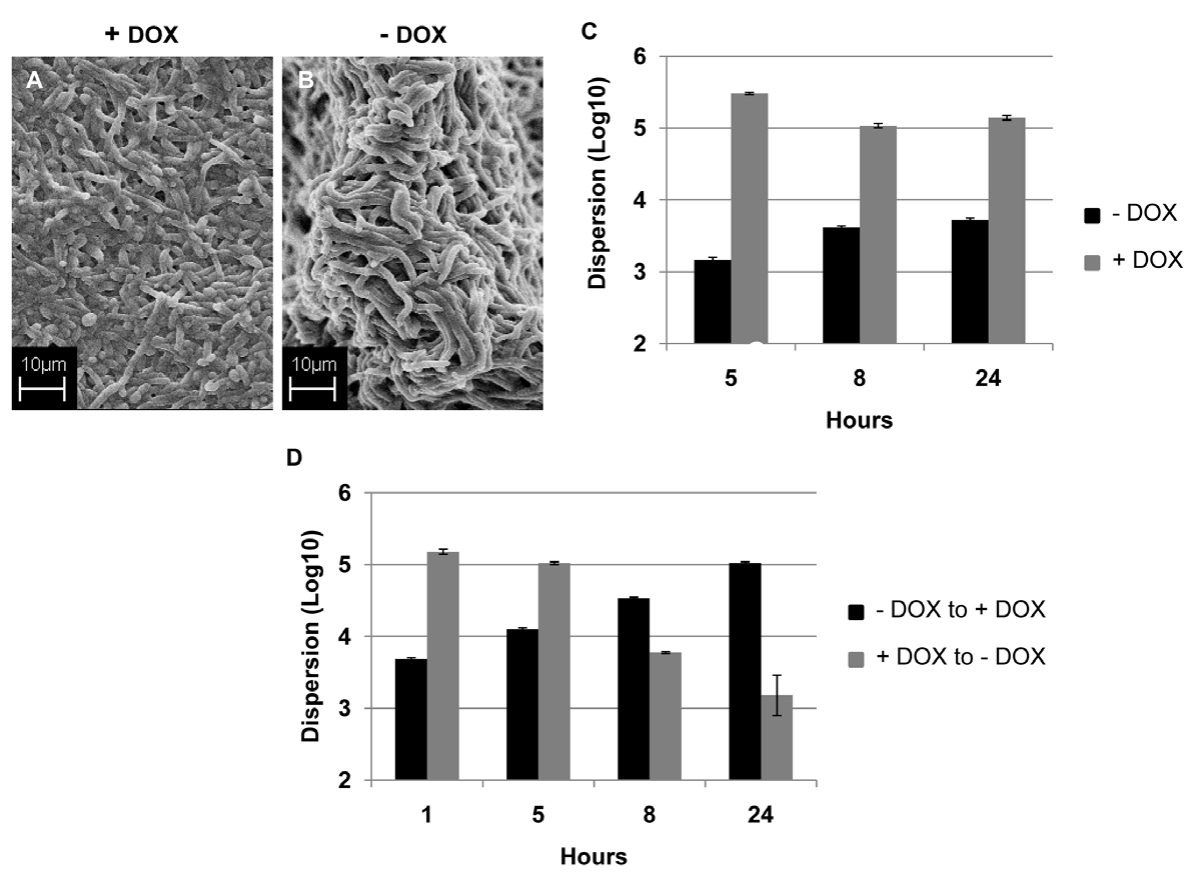
Regulation of *C. albicans* biofilm dispersion by *UME6*. Biofilms developed by the *C. albicans tetO-UME6* strain in the presence (A) and absence (B) of DOX were imaged by SEM. Number of dispersed cells released from biofilms formed by the *tetO-UME6* strain in the presence and absence of DOX were enumerated (C) and so was the impact of media switch from + DOX to - DOX and vice versa, on biofilm dispersion (D).

As mentioned before, *C. albicans* biofilms contain a mixture of yeast, hyphal and pseudohyphal morphologies, but dispersed cells are mostly in the yeast form. This seems to indicate an important role for morphogenetic conversions not only in the process of biofilm formation, but also during biofilm dispersion. For these sets of experiments we took advantage of the availability of the genetically engineered strains of *C. albicans* (*tetO-UME6* and *tetO-PES1*) in which levels of gene expression and morphogenetic conversions can be externally manipulated simply by addition or omission of doxycycline in the medium [34],[35]. *UME6* encodes a recently identified filament-specific transcriptional regulator important for *C. albicans* hyphal extension [43], whereas *PES1* encodes the pescadillo homolog in *C. albicans* which is critical for the reverse morphological transitions (from filament to yeast) and for lateral budding [35].

ppat.1000828.In.tifthe presence of DOX, the strain containing one allele of *UME6* under control of the *tet* operator (*tetO-UME6*) developed biofilms comprised mostly of yeast and filamentous morphologies, although with a lower percentage of true hyphal elements (Figure 5A). In contrast, this strain proliferated into a highly filamentous biofilm when grown in media in the absence of DOX, when *UME6* is expressed at high constitutive levels (Figure 5B). Measurement of biofilm dispersion from both types of biofilms revealed that overtime, the biofilms grown in the presence of DOX released at least 150–200 times more yeast cells than the highly filamentous biofilms formed in the absence of the antibiotic (Figure 5C). When 24 hr old biofilms grown in the presence of DOX were switched to medium without DOX, there was a gradual decrease in the number of dispersed cells and by 24 hr after the switch, the extent of biofilm dispersion was reduced by approximately 85% (Figure 5D). Microscopy also revealed that under these conditions the biofilm characteristics had changed from the mostly yeast/pseudohyphal to the mostly hyphal architecture (data not shown). An opposite effect was observed when DOX was added to 24 hr biofilms which had initially been grown in the absence of the antibiotic. A significant increase (approximately 3-fold) in biofilm dispersion was observed as early as 5 hr after the switch, and by 24 hr the level of biofilm dispersion had increased by 20-fold (Figure 5D). Addition of DOX triggered the production (and eventual release) of yeast/pseudohyphae from the originally highly filamentous biofilm grown in the absence of the antibiotic.

**Figure 6:**
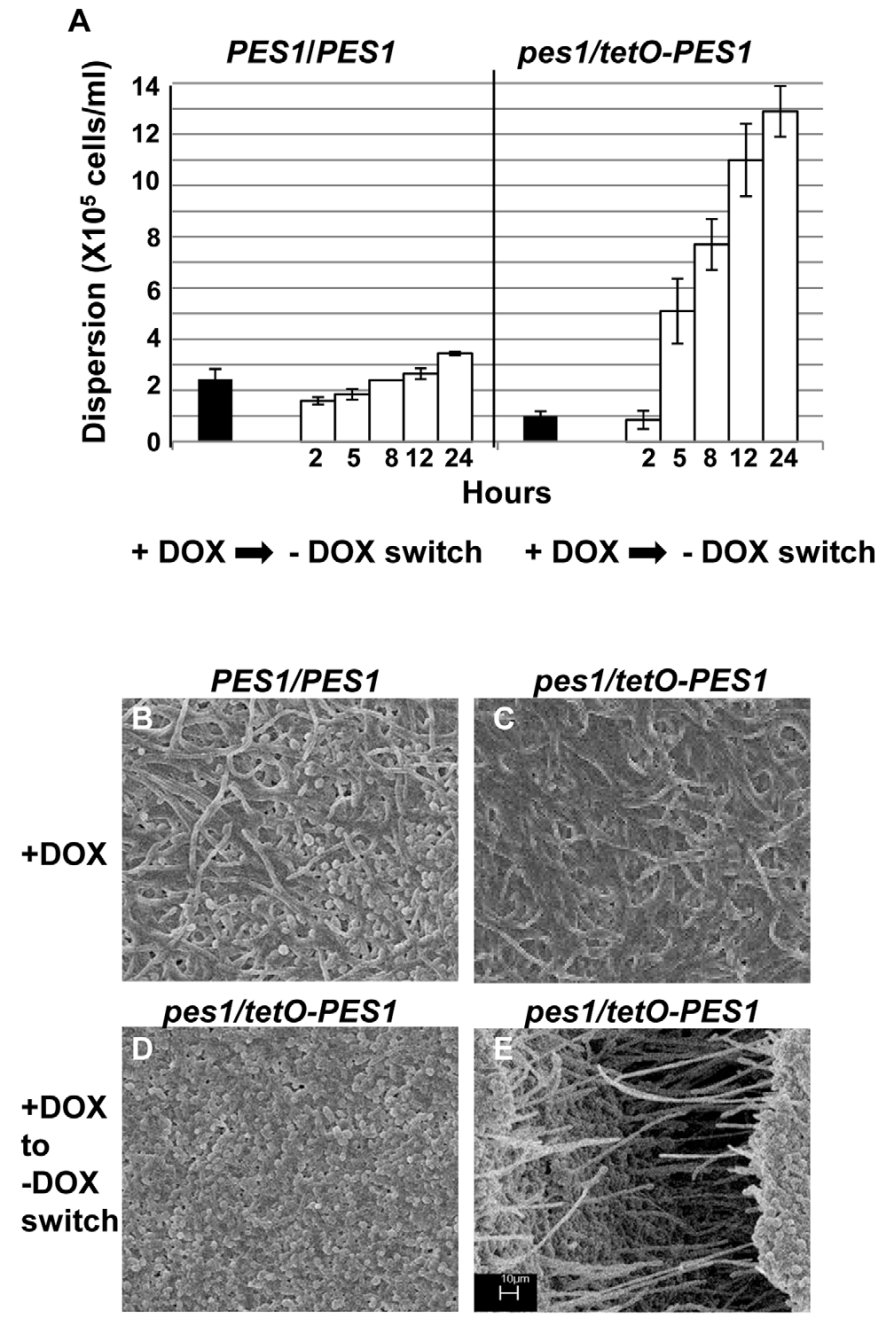
Regulation of *C. albicans* biofilm dispersion by *PES1*. Biofilms (formed by the *C. albicans PES1/PES1* and *tetO*-*PES1* strains) were developed for 17 hours in YNB with DOX, and the number of dispersed cells counted. DOX was withdrawn from the media and number of dispersed cells counted at various time points after antibiotic removal (A). Biofilms formed by the *C. albicans PES1/PES1* and *tetO*-*PES1* strains were developed in the presence of DOX (B and C respectively). Impact of change DOX withdrawal on the *tetO*-*PES1* biofilm is shown in panel D. Panel E shows the morphology of the cells in a crack in the *tetO*-*PES1* biofilm developed after removal of the antibiotic from the medium. Since *PES1/PES1* showed similar biofilm phenotype before and after media change, only one representative image for both conditions is shown. Scale bar for SEM corresponds to 10 µm for all four images.

Hyphal and pseudohyphal filaments constitutively produce yeast cells on their subapical segments, referred to as lateral yeast cells. Since, *C. albicans* cells dispersed from biofilms were primarily in the yeast form we wanted to investigate the significance of lateral yeast cells that could provide additional insights into the molecular mechanisms of biofilm dispersal. We employed a genetically engineered strain of the recently described *C. albicans* pescadillo homolog (*PES1*) that functions in the production of lateral yeast growth on filamentous cells [35]. In this strain, one allele of *PES1* is placed under the control of the same tetracycline-regulatable promoter so that production of lateral yeast cells from hyphae can be manipulated by omitting or adding DOX from the growth medium. *C. albicans tetO-PES1* and the wildtype strain *PES1/PES1* were allowed to develop biofilms in medium containing DOX. Total numbers of cells released from the biofilms were enumerated at this time point. After 17 hr of biofilm growth, DOX was withdrawn from the medium and the biofilm was left to continue growing in the absence of the antibiotic. At various time points after the medium switch (2, 5, 8, 12, 24 hr) cell numbers and viability of cells dispersed from biofilms were quantified. Even before the medium switch, the extent of dispersal from the *tetO*-*PES1* biofilms was 2 times lower than the dispersal from wild type biofilms (Figure 6A). However, after DOX was withdrawn from the growth medium (leading to *PES1* overexpression), there was a drastic increase in the number of dispersed cells beginning at 5 hr (Figure 6A). SEM images of the 17 hr biofilms grown in the presence of DOX, showed a typical wildtype biofilm containing all the major morphological forms of *C. albicans* (Figure 6B). On the other hand, under the same conditions the *tetO*-*PES1* biofilms were mostly in the filamentous form (Figure 6C), indicating a reduction in the number of lateral yeast cells due to DOX-mediated depletion of *C. albicans* Pes1p. In the absence of DOX, leading to an overexpression of Pes1p, these filaments induced high quantities of lateral yeast cells on the topmost layers of the biofilm (Figure 6D). The presence of lateral yeast cells is clearly evident from imaging a crack in the biofilm biomass, revealing several hyphae under a blanket of lateral yeast cells (Figure 6E).

## Discussion

Formation of biofilms allows microbial pathogens to create a safe sanctuary in which sessile cells remain in a protected environment. However, cells within a biofilm may be also confronted with adverse environmental conditions (*i.e.* reduced nutrient availability, accumulation of toxic waste products) so that dispersion of cells would be beneficial for survival. Furthermore, this release of cells from the original biofilm community is required to generate novel communities at new locations. It follows that gaining knowledge about mechanisms regulating biofilm dispersion, at both the physiochemical and molecular levels can potentially lead to better strategies for the prevention and treatment of biofilm-associated infections. This is particularly important in the case of *C. albicans*, since dispersed cells are responsible for candidemia and disseminated invasive candidiasis, which are among the gravest forms of infection and carry the highest mortality rates [11], [44]–[46].

Biofilm formation by *C. albicans* progresses through multiple development stages, beginning with attachment to a surface, microcolony formation, proliferation and development and maturation involving encapsulation within an exopolymeric matrix [21],[47]. Cells at each stage of the biofilm developmental process display distinct phenotypes and properties markedly different from those of the same group growing planktonically. The developmental life cycle of *C. albicans* biofilms comes full circle when biofilm cells disperse and go on to colonize new surfaces again. In bacteria, loss of cells from biofilms is known to be triggered by changes in the physiochemical environment, including changes in nutrients, starvation, quorum sensing, modifications in exocellular biofilm components and regulation at the molecular level of certain gene products [26]–[29],[31],[48],[49]. In *C. albicans*; however, very little is known about the environmental cues that initiate biofilm dispersion, about the phenotypic properties of the dispersed cells, or about the molecular mechanisms governing release of cells from biofilms. In the present study, we investigated these aspects of this understudied stage of the *C. albicans* biofilm life cycle.

We took advantage of a simple *in vitro* model of *C. albicans* biofilm formation under conditions of flow recently described by our group [20] which enabled us to develop *C. albicans* biofilms under controlled conditions of media flow for prolonged periods of time. We were thus able to study the phenomenon of biofilm dispersion in a controlled manner which is not possible by using “static” biofilm models such as the widely used 96-well microtiter plate model [19],[50]. At the same time, one of the decisive advantages of this model is that it yields enough dispersed cells needed for all subsequent phenotypic and molecular analyses. Furthermore, our *in vitro* flow model can simulate physiologically relevant shear stresses experienced by the biofilms in the blood vessel.

Rather than an often hypothesized end-stage process, we found that dispersion occurred continuously over the course of biofilm development. Our initial observations indicated that, regardless of the media used, the number of dispersed cells released into the flow-through liquid depended upon the growth stage of the biofilm. During the early stages of biofilm growth (0–3 hr), the extent of dispersion is lesser than that at the intermediate stage of biofilm growth (5–12 hr). As the biofilms matured (20–24 hr), the numbers of dispersed cells again decreased. Because we grow our biofilms under conditions of flow, the low levels of dispersion observed during the early stages of biofilm development may be due to passive dispersal of biofilms as a result of hydrodynamic parameters such as shear stress, a phenomenon also referred to as “erosion” [51],[52]. Overall, these observations carry important clinical implications as release of cells from an *in vivo* biofilm (*i.e.* catheter) may occur early after initial colonization of the biomaterial without the need for the formation of a fully developed mature biofilm. Maximum numbers of cells are released when the biofilm proliferates rapidly during the intermediate phase (5–12 hr), while numbers decrease again during the stationary phase of a mature biofilm. As long as biofilms were supplied with fresh nutritients, complete cessation of dispersion was never observed, even after 48 hr of growth.

Since, *C. albicans* biofilms are made up of a mixture of yeast, pseudohyphae and hyphae [10],[21],[22], we hypothesized that the cells released from biofilms may also include all three morphological forms. To the contrary, we found that under a steady rate of media flow, the dispersed cells occurred predominantly in the yeast form. Interestingly, we observed that a large percentage of these yeast cells were unbudded and elongated in shape, depending on the nutrient content of the media used for biofilm development. Larger numbers of unbudded cells were recovered from biofilms grown in synthetic defined media (YNB and RPMI) rather than from nutrient rich medium like YPD. Although a quantitative distribution of the total dispersed cell population was not performed in this study, we have found that the majority of the dispersed cells originate from the topmost outer layers of the biofilm (not shown). The yeast form of *C. albicans* is considered to be most conducive for dissemination into the blood stream [41],[53]; and the results of our study may serve as a proof of principle that release of these potentially infectious particles from biofilms may prove to be a major mechanism of candidemia and disseminated invasive infections that often occur after *C. albicans* biofilms initially form on the surface of intravascular catheters.

In bacteria the nutritional status of the environment most often dictates biofilm dispersal and both decreases and increase in nutrients have been described to induce biofilm dispersion [26],[27],[33]. In the case of *C. albicans*, we also observed that composition of the media used for biofilm formation affected dispersion: in general, the richer the medium, the greater were the numbers of dispersed cells in the flow-through liquid. For example, the hierarchy of media triggering dispersion was YPD > YNB >RPMI. Detachment (rather than dispersion) of whole *C. albicans* biofilms under the influence of rich medium has recently been reported by Sellam *et. al* [33]. However for their study, the investigators used a strong hydrodynamic force (more than 17.3 dynes/cm^2^), forcing displacement of the biofilms from the surface of the tubing. Our study employed a steady, laminar flow of 1 ml/min (corresponding to a shear force of 4.52 dyn/cm^2^) to study biofilm dispersion. This mechanical force is physiologically relevant since the normal time-averaged levels of venous stresses range between 1–5 dyne/cm^2^ [54]. A similar correlation between biofilm dispersion and flow rate has been previously reported by Baillie and Douglas: in their study biofilms grown on cellulose membrane subjected to a flow rate of 1 ml/min of YNB yielded biofilm dispersion rates similar to those observed in our study under similar conditions of flow [55]. Furthermore, we discovered that the rate and extent of biofilm dispersion was greatly influenced by the availability of carbon sources. We found that increasing amounts of glucose in the growth medium increased the levels of biofilm dispersion, so much so that 500 mM glucose initiated biofilm dissolution after 20 hr. In many bacterial species capable of forming biofilms, availability of nutrients especially high glucose concentrations is known to suppress biofilm formation by increasing biofilm dispersion [56],[57]. Our findings are consistent with earlier findings by Sauer *et. al*, who demonstrated biofilm dissolution in *Pseudomonas aeruginosa* by high concentrations of carbon sources [27]. Massive dispersion under favorable growth conditions seem to indicate a biofilm survival strategy whereby *C. albicans* cells only benefit from the biofilm mode of growth at low nutrient concentrations, but abandon this mode of growth when conditions in the environment become favorable. No alternative carbon source used in the present study (galactose or maltose) was as effective as glucose in induction of biofilm dispersion. In fact, the rate of dispersion was reduced >50-fold when sterile PBS instead of media was supplied to mature biofilms, to induce starvation. Until now, in the field of *C. albicans* biofilm research, it was speculated that nutrient depletion enables production and release of cells from the biofilms thereby increasing dispersion [22]. Although this may still hold true for statically grown biofilms (in fact we have observed massive detachment events as static biofilms age over 72 and 96 hr) our results using the more physiological flow model show the opposite: we found that poor nutrient sources led to significant curtailment in the release of cells from a biofilm while media rich in nutrients triggered higher rates of biofilm dispersion.

In addition to nutritional composition, we observed that the pH of the growing medium also exerts an important effect on *C. albicans* biofilm dispersion, which was enhanced at acidic pH and decreased under alkaline conditions (Figure S2A). We speculate that acidic pH conditions induce lateral yeast formation from biofilm hyphae and also increase the number of yeast cells in the biofilm, thereby resulting in greater number of cells dispersed under flow. Treatment of preformed biofilms with farnesol, a quorum sensing molecule that inhibits *C. albicans* filamentation and biofilm formation [39],[40] only led to a significant increase in dispersion when supraphysiological concentrations of this compound were used (Figure S2B). Although we cannot disregard the effect of accumulating farnesol concentrations in the detachment events observed when biofilms are grown under static conditions, it is likely that the continuous flow conditions used in this study, effectively preventing accumulation of high farnesol concentrations, are responsible for the lack of effect on biofilm dispersion observed at lower (physiological) concentrations of farnesol. The same diluting effect of continuous flow can also explain the lack of effect on biofilm dispersion observed at physiological concentrations of tyrosol (results not shown).

In order to colonize distal sites, cells released from biofilms must be able to disperse into the host environment and adhere to and damage the endothelial cells lining blood vessels before entering the tissues [41]. Hence we postulated that the infectious particles released from *C. albicans* biofilms may possess enhanced adhesive and invasive properties. Indeed, we found that *C. albicans* yeast cells dispersed from 37°C grown, 24 hr old biofilms were at least 35–40% more adherent to polystyrene compared to age and temperature matched planktonic yeast cells. Not only were the dispersed cells more adherent, but they also developed germ tubes in numbers consistently higher than planktonic cells. This result was interesting because it meant that cells dispersed from *C. albicans* biofilms indeed were infectious particles, already ready to deploy at least two of the major virulence factors considered of critical importance during the pathogenesis of candidiasis [58]–[61]. This also seems to indicate that free living; highly adherent dispersed cells had prospects of colonizing and establishing biofilms at newer sites and indeed, we found that the dispersed cells could develop more robust biofilms compared to planktonic cells. Armored with properties such as better adhesion and filamentation, it was not surprising that dispersed cells from *C. albicans* biofilm also display enhanced adhesion to endothelial cells and cause increased endothelial cell damage, which represent major hallmarks of the infectious process. But perhaps, the ultimate test regarding virulence is the assessment of pathogenesis in the murine model of hematogenously disseminated candidiasis. Indeed, all the above characteristics possessed by the dispersed cells translated to the *in vivo* scenario, where cells dispersed from biofilms demonstrated enhanced virulence compared to their planktonic counterparts. This finding seems to indicate that dispersed cells retain their higher virulence over several generations, which raises the question whether heritable epigenetic modifications are responsible for enhanced adhesion, filamentation and virulence of dispersed cells.

Studying the mechanisms that regulate biofilm dispersion has been an area of intense activity in the field of bacterial biofilms. Several attempts have been made to identify cellular responses that contribute to the occurrence of active biofilm dispersion in pathogenic bacteria. *Streptococcus mutans* enables biofilm dispersion by releasing enzymes that break down polysaccharide matrix material [62]. In *P. aeruginosa*, *P. putida* and *Shewanella oneidensis* changes in oxygen, nutrient sources and several chemical parameters have been reported to induce dispersion [27],[63],[64]. Dispersion in *Escherichia coli* is mediated largely through the collective regulation of intracellular glycogen biosynthesis and central carbon metabolism, alteration of matrix components, activation of flagellum, and motility [65]–[67]. Thus it is becoming clear that dispersion from mature biofilms maybe triggered by environmental cues that coincide with specific phenotypic changes in the organism.

In this study, we demonstrate that *C. albicans* biofilm dispersion is regulated to a great extent by two important morphogenetic processes; first, the ability to undergo morphogenetic conversions and second, the ability of filaments to produce lateral yeast cells. For the first study, we utilized a genetically engineered strain of *C. albicans*, in which one allele of Ca*UME6*, a positive regulator of filamentation is placed under control of a tet-regulatable promoter [34]. The extent of dispersal of biofilm cells was greatly dependent on the morphological characteristics of the biofilm. An all hyphal biofilm (medium without DOX; *UME6* overexpression) released fewer number of cells compared to a mostly pseudohyphal and yeasty biofilm (medium with DOX, *UME6* repression). We showed that *C. albicans* biofilm dispersion could be controlled to a large extent by controlling the morphology of this fungus. This knowledge could be of great importance in designing antifungal drugs that could curtail biofilm dispersion by targeting specific morphological forms in biofilms. For example, the HSP90 inhibitor geldanamycin (GDA) induces hyperfilamentation in *C. albicans* yeast cells and thereby renders the cells highly avirulent in a mouse model [68]. More importantly, in combination with fluconazole, GDA is fungistatic in planktonic cells [69],[70]. Thus, development of drugs like GDA could prove extremely potent in abolishing dispersion of cells from biofilms.

Microscopic images in this study revealed that several dispersed cells appeared to be released from the upper-most hyphal layers of the *C. albicans* biofilms. This observation was confirmed when we showed that increasing lateral yeast cells in biofilms by overexpression of Ca*PES1* [35] increased the extent of biofilm dispersion. SEM showed an overwhelming presence of yeast cells in the topmost layers of the biofilm, and a crack in the biofilm disclosed that these indeed were lateral yeast cells produced by the hyphae now completely covered by them. The repression of Ca*PES1* abolished lateral yeasts from the topmost layers of the biofilm resulting in a decrease in numbers of dispersed cells and reemergence of the hyphal mesh. Depletion of the levels of the Pes1p corresponding with a decrease in lateral yeasts is known to render *C. albicans* avirulent in *Galleria mellonella* model [35]. Discovery of antifungal agents targeting Pes1p has implications for *C. albicans* biofilm related infectious diseases because lateral yeasts released from biofilms have the highest potential to disseminate and cause invasive diseases. In *S. cerevisiae*, the *pescadillo* homolog Yph1 participates in a nutritional signaling network that relays information about the cell's nutritional status to both the cell cycle machinery and the ribosomal biogenesis machinery [71]. In *C. albicans*, fresh media has been shown to induce Pes1p expression and subsequent increase in lateral yeast production [35]. If *C. albicans* Pes1p is linked to a nutritional signaling network, it could very well be a major player in orchestrating biofilm dispersion via interactions with the cAMP-dependent protein kinase (PKA) and TOR pathways. Thus, the *C. albicans* pescadillo homolog may represent a link between two processes with important roles in biofilm dispersion: morphology and sensing of nutritional conditions.

In summary, we have initiated studies on the phenomenon of *C. albicans* biofilm dispersion, facilitated by the recent development of a simple model for biofilm formation under physiological conditions of flow. In contrast to a “massive” detachment event, our results indicate that dispersal of cells, which are mostly in the yeast form, occurs continuously throughout the biofilm developmental cycle. The dispersion process is dependent on different environmental factors, including nutrition and pH. Dispersed cells from biofilm display distinct phenotypic properties associated with increased virulence. In addition, our results point to the presence, at the molecular level, of a complex regulatory circuitry that orchestrates the phenomenon of biofilm dispersion in *C. albicans*.

## Supporting Information

Figure S1 Morphology of cells dispersed from *C. albicans* biofilms. *C. albicans* biofilms were grown for 24 h in RPMI medium. (A) Light microscopy of cells released by biofilms, (B) Light microscopy of yeast cells appear to be budding off the biofilm hyphae. (C) Scanning electron microscopy of biofilm topmost layer showing yeast cells budding off biofilm hyphae. Scale bars are 10 µm for all panels.
(3.44 MB PPT)

Figure S2 Effect of media pH and farnesol treatment on biofilm dispersion rates. Biofilms were developed for 24 h in YNB medium pH 7.2. The media pH was then increased (to pH 9) or decreased (to pH 3) and effects of this change on recovery of dispersed cells were quantified at different times after the change in pH (A). Biofilms were also treated with 30 µM, 100 µM or 300 µM of farnesol and the changes in biofilm dispersion rates were quantified at various time points (B).
(0.15 MB PPT)

Table S1 Quantitative segregation of dispersed cells based on budding patterns and morphology in the three different media
(0.12 MB PPT)

Table S2 Quantitative segregation of planktonic and dispersed cells adhered to endothelial cells based on their morphological differentiation upon adhesion
(0.13 MB PPT)
