# Supplementary material for: Dispersion as an Important Step in the Candida albicans Biofilm Developmental Cycle
Source: PLoS Pathog. 2010 Mar 26;6(3):e1000828. doi: 10.1371/journal.ppat.1000828 (PMC2847914; doi:10.1371/journal.ppat.1000828)
Supplement: Figure S1 — Morphology of cells dispersed from C. albicans biofilms. C. albicans biofilms were grown for 24 h in RPMI medium. (A) Light microscopy of cells released by biofilms, (B) Light microscopy of yeast cells appear to be budding off the biofilm hyphae. (C) Scanning electron microscopy of biofilm topmost layer showing yeast cells budding off biofilm hyphae. Scale bars are 10 µm for all panels. (3.44 MB PPT) [file ppat.1000828.s001.ppt]

## Slide 1
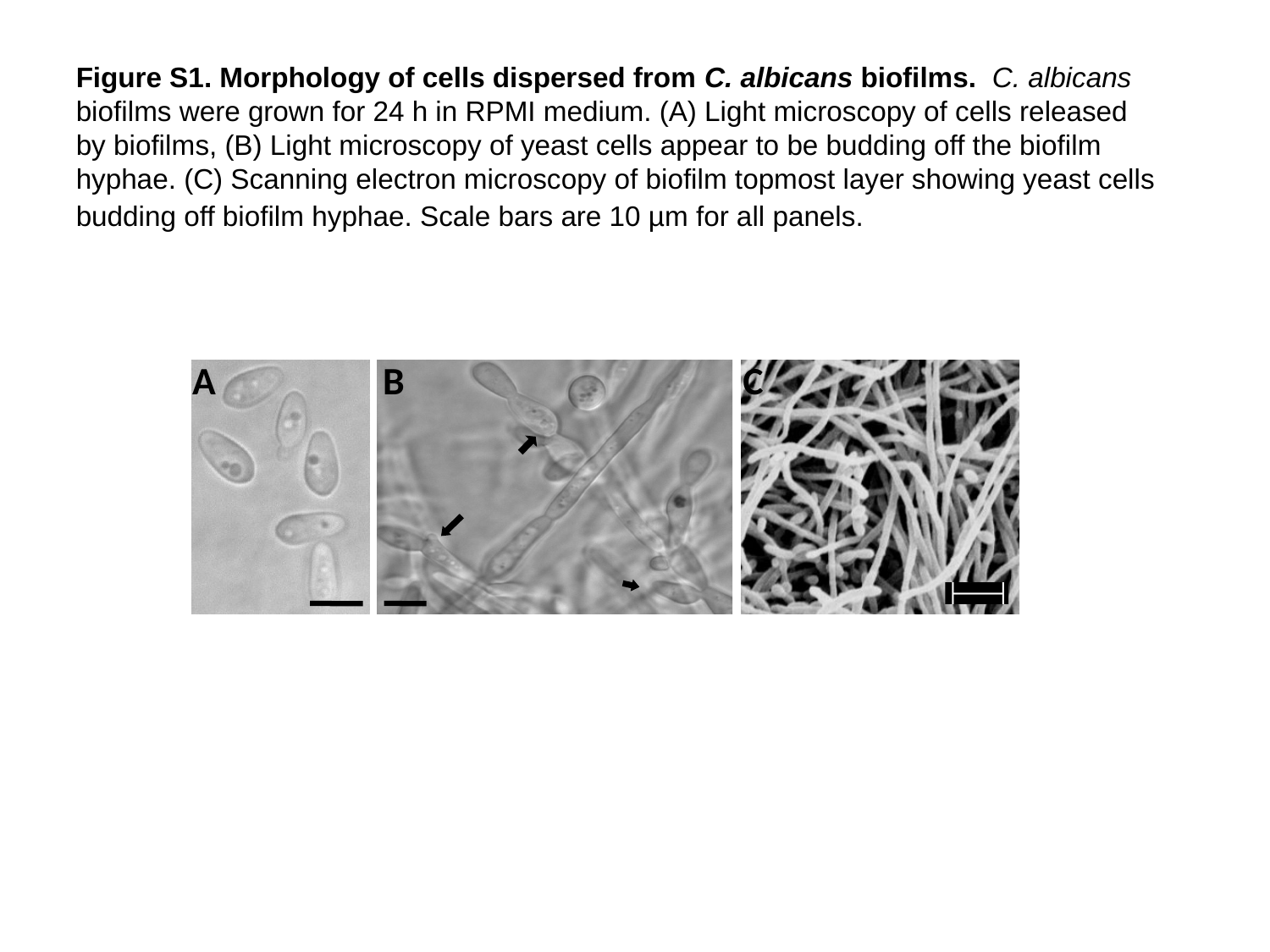

Figure S1. Morphology of cells dispersed from C. albicans biofilms. C. albicans biofilms were grown for 24 h in RPMI medium. (A) Light microscopy of cells released by biofilms, (B) Light microscopy of yeast cells appear to be budding off the biofilm hyphae. (C) Scanning electron microscopy of biofilm topmost layer showing yeast cells budding off biofilm hyphae. Scale bars are 10 µm for all panels.
A
C
B
