# Supplementary material for: Dispersion as an Important Step in the Candida albicans Biofilm Developmental Cycle
Source: PLoS Pathog. 2010 Mar 26;6(3):e1000828. doi: 10.1371/journal.ppat.1000828 (PMC2847914; doi:10.1371/journal.ppat.1000828)
Supplement: Figure S2 — Effect of media pH and farnesol treatment on biofilm dispersion rates. Biofilms were developed for 24 h in YNB medium pH 7.2. The media pH was then increased (to pH 9) or decreased (to pH 3) and effects of this change on recovery of dispersed cells were quantified at different times after the change in pH (A). Biofilms were also treated with 30 µM, 100 µM or 300 µM of farnesol and the changes in biofilm dispersion rates were quantified at various time points (B). (0.15 MB PPT) [file ppat.1000828.s002.ppt]

## Slide 1
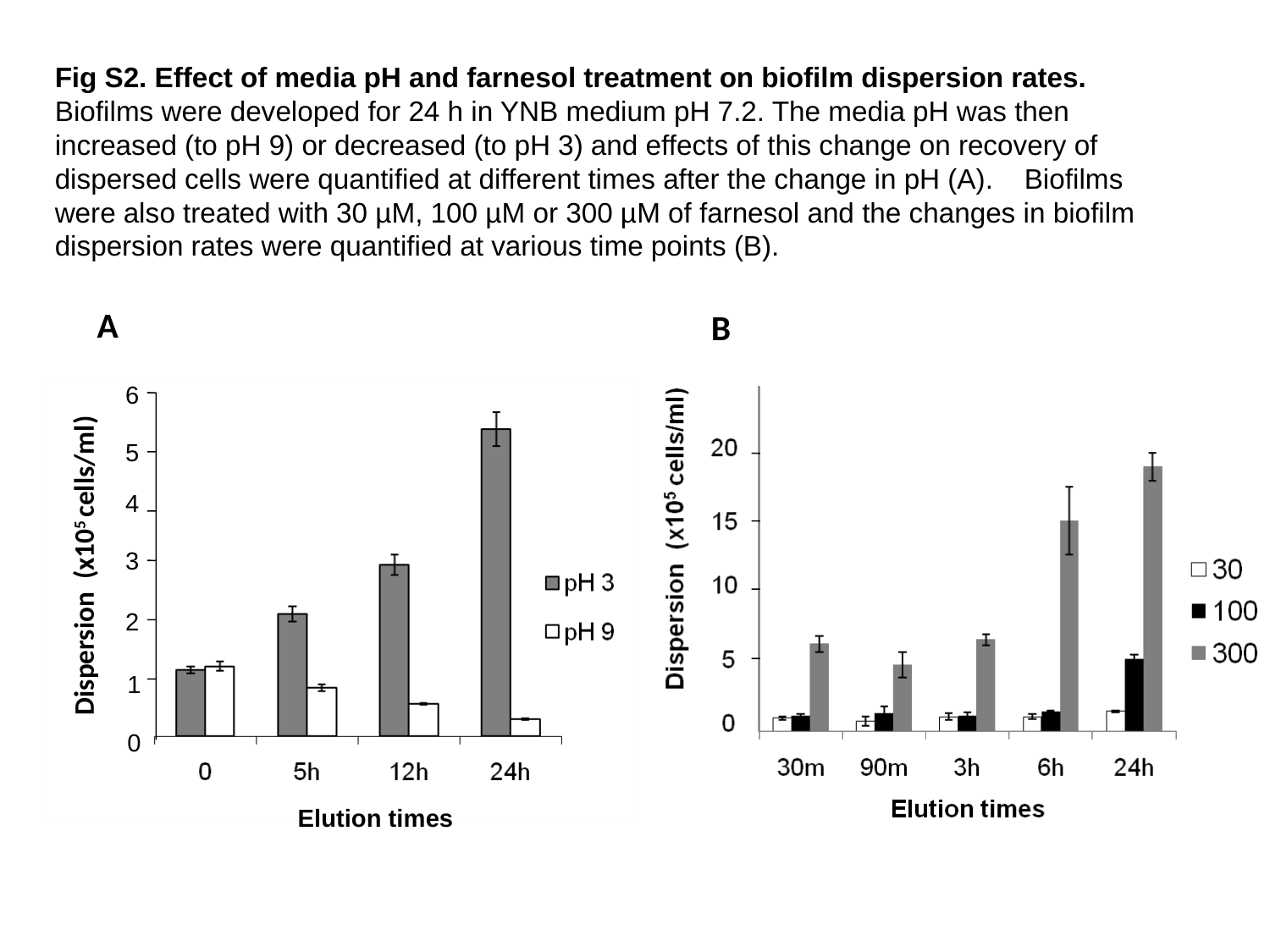

Fig S2. Effect of media pH and farnesol treatment on biofilm dispersion rates. Biofilms were developed for 24 h in YNB medium pH 7.2. The media pH was then increased (to pH 9) or decreased (to pH 3) and effects of this change on recovery of dispersed cells were quantified at different times after the change in pH (A). Biofilms were also treated with 30 µM, 100 µM or 300 µM of farnesol and the changes in biofilm dispersion rates were quantified at various time points (B).
A
B
6
5
4
3
2
1
0
Dispersion (x105 cells/ml)
Elution times
