# Supplementary material for: Dispersion as an Important Step in the Candida albicans Biofilm Developmental Cycle
Source: PLoS Pathog. 2010 Mar 26;6(3):e1000828. doi: 10.1371/journal.ppat.1000828 (PMC2847914; doi:10.1371/journal.ppat.1000828)
Supplement: Table S1 — Quantitative segregation of dispersed cells based on budding patterns and morphology in the three different media (0.12 MB PPT) [file ppat.1000828.s003.ppt]

## Slide 1
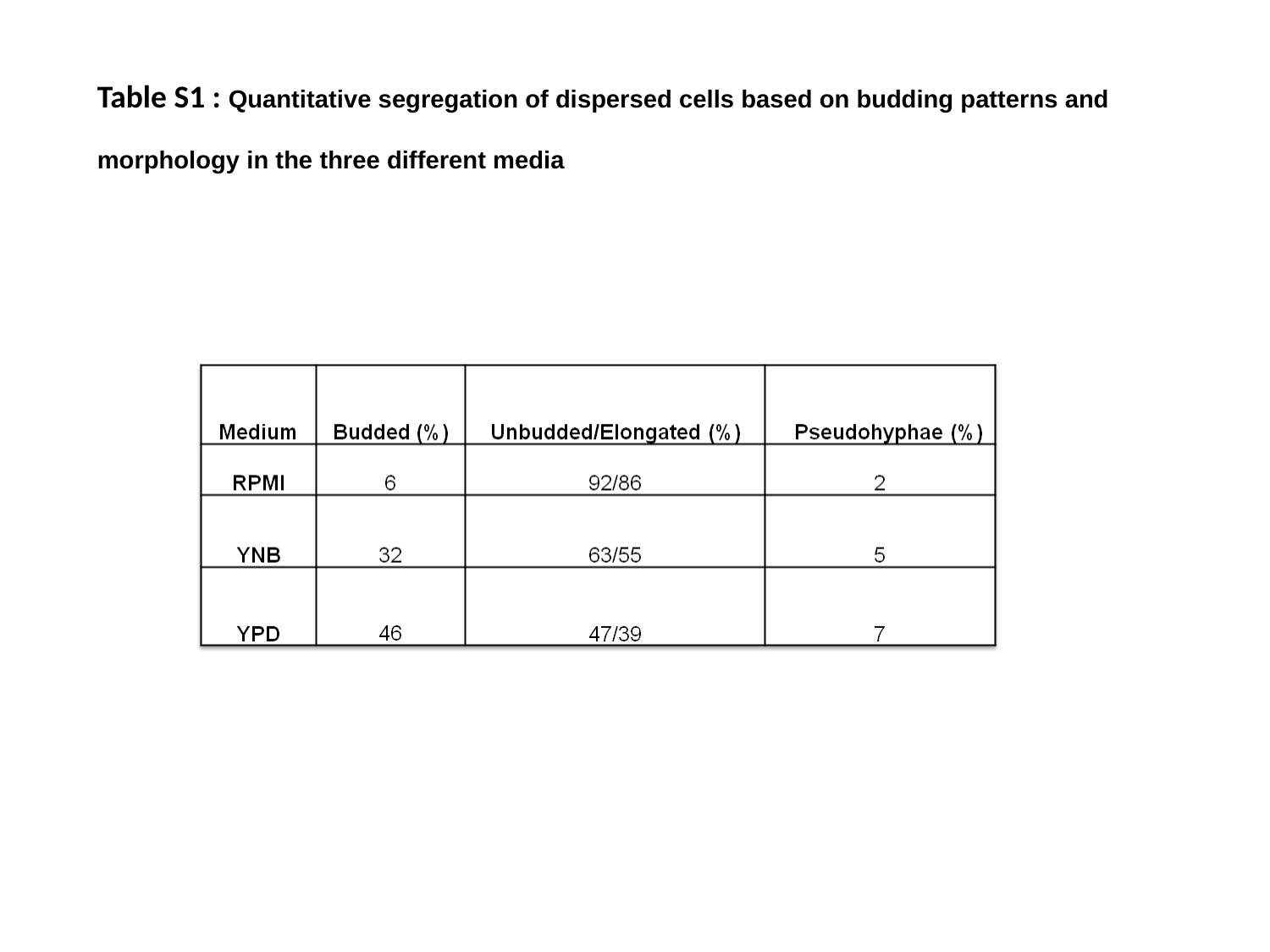

Table S1 : Quantitative segregation of dispersed cells based on budding patterns and morphology in the three different media
