# Supplementary material for: Dispersion as an Important Step in the Candida albicans Biofilm Developmental Cycle
Source: PLoS Pathog. 2010 Mar 26;6(3):e1000828. doi: 10.1371/journal.ppat.1000828 (PMC2847914; doi:10.1371/journal.ppat.1000828)
Supplement: Table S2 — Quantitative segregation of planktonic and dispersed cells adhered to endothelial cells based on their morphological differentiation upon adhesion (0.13 MB PPT) [file ppat.1000828.s004.ppt]

## Slide 1
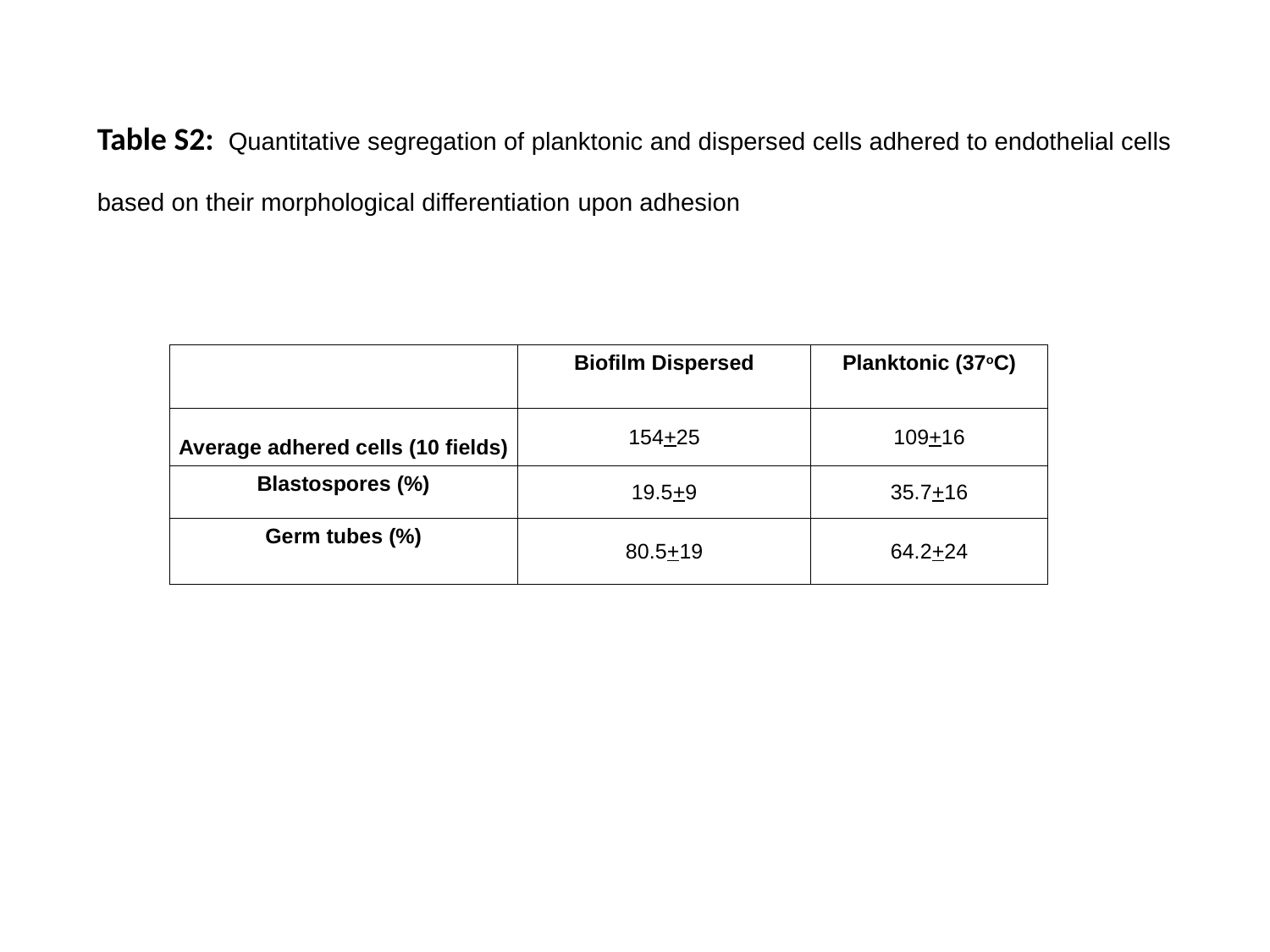

Table S2: Quantitative segregation of planktonic and dispersed cells adhered to endothelial cells based on their morphological differentiation upon adhesion
| | Biofilm Dispersed | Planktonic (37oC) |
| --- | --- | --- |
| Average adhered cells (10 fields) | 154+25 | 109+16 |
| Blastospores (%) | 19.5+9 | 35.7+16 |
| Germ tubes (%) | 80.5+19 | 64.2+24 |
